# BMI and idiopathic infertility drive polarized redox phenotypes in human follicular fluid

**DOI:** 10.3389/fendo.2026.1890028

**Published:** 2026-07-21

**Authors:** Katarzyna Olszak-Wąsik, Andrzej Tukiendorf, Rafał Kurzawa, Iwona Kulik-Parobczy, Ayla Sargın, Aleksandra Kasperczyk

**Affiliations:** 1 Department of Gynaecology, Obstetrics and Oncological Gynaecology, Bytom, Poland; 2 Department of Gynaecology, Obstetrics and Oncological Gynaecology, Faculty of Medical Sciences in Zabrze, Zabrze, Poland; 3 The Fertility Partnership (TFP) Fertility Macierzyństwo, Kraków, Poland; 4 TFP Fertility Vitrolive, Szczecin, Poland; 5 Opole University of Technology, Faculty of Physical Education and Physiotherapy, Opole, Poland; 6 Department of Gynecology and Reproductive Health, Pomeranian Medical University, Antalya, Poland; 7 IVF Department, Antalya Life Hospital (Yaşam Hastanesi), Antalya, Türkiye; 8 Medical University of Silesia, Poland; Department of Biochemistry, Faculty of Medical Sciences in Zabrze, Zabrze, Poland

**Keywords:** Bayesian inference, follicular fluid, hierarchical clustering, idiopathic infertility, *in vitro* fertilization, obesity, oxidative stress, redox polarization

## Abstract

**Background:**

Oxidative balance within follicular fluid (FF) plays an important role in oocyte competence and female fertility. However, most previous studies have focused on isolated oxidative biomarkers rather than on the coordinated organization of the follicular redox system. This study aimed to identify system-level oxidative phenotypes within FF and evaluate their associations with infertility-related clinical and metabolic factors.

**Methods:**

Seventy-seven women undergoing IVF treatment were included. Nineteen oxidative and antioxidative biomarkers were measured in FF collected separately from both ovaries. Bayesian multivariable regression with Markov chain Monte Carlo inference was combined with directional indicator analysis and unsupervised taxonomic clustering to identify latent oxidative phenotypes.

**Results:**

Two structurally distinct and antagonistically organized oxidative phenotypes were identified within FF. Phenotype 1 comprised biomarkers representing the primary antioxidant defense system, whereas Phenotype 2 included enzymes involved predominantly in secondary detoxification pathways. Idiopathic infertility and BMI demonstrated significant and opposite directional effects across the two phenotypes. Specifically, increasing BMI and idiopathic infertility were associated with decreased activity of biomarkers belonging to Phenotype 1 and increased activity of biomarkers assigned to Phenotype 2 (p = 0.0177 and p = 0.0097, respectively).

**Conclusions:**

The follicular redox environment demonstrates a polarized and system-level biochemical architecture rather than uniform oxidative alterations. BMI and idiopathic infertility appear to selectively reorganize oxidative pathways within FF through opposite effects on two antagonistically structured phenotypes. These findings suggest a shared pathophysiological mechanism reorganizing the follicular antioxidant network in both conditions and challenge the single-biomarker paradigm in reproductive oxidative stress research.

## 1 Introduction

Infertility represents a growing global health challenge, with recent large-scale analyses indicating a steady increase in prevalence among women and men of reproductive age worldwide (1). Although many factors contributing to infertility have been identified and characterized, a substantial proportion of patients continues to experience unsatisfactory treatment outcomes despite advances in assisted reproductive technology (2). In particular, a significant subset of couples receives a diagnosis of idiopathic infertility, defined as failure to achieve pregnancy despite comprehensively normal clinical and laboratory findings in both partners (2, 3). This diagnosis implies the presence of underlying biological mechanisms that remain undetected by standard diagnostic approaches. Approximately 30% of infertile couples fall into this category, which constitutes a diagnosis of exclusion and reflects the limitations of current diagnostic methodologies rather than a true absence of pathology (2).

Follicular fluid (FF) is the biological medium surrounding the oocyte within the antral follicle and plays a central role in oocyte growth and maturation. FF represents a specialized microenvironment integrating systemic and local regulatory processes that determine oocyte competence (4). It contains hormones, cytokines, metabolites, and a wide array of oxidative and antioxidative components that reflect the redox status of the follicular environment (5). Such coordinated interactions raise the possibility that oxidative processes within follicular fluid may organize into biologically distinct and potentially opposing biochemical response patterns. Its oxidative and antioxidative components reflect the redox status of the follicular environment and provide a direct functional representation of follicular physiology (6, 7).

Redox homeostasis within the follicular environment represents a highly coordinated and dynamic system, rather than a collection of independent biochemical markers (8, 9). Reactive oxygen species (ROS), generated as by-products of cellular metabolism — primarily through mitochondrial oxidative phosphorylation — play essential physiological roles in ovarian function (9, 10). At physiological concentrations, ROS regulate key processes involved in folliculogenesis, ovulation, and oocyte maturation (11, 12). In addition, ROS participate in intracellular pathways involved in fertilization and early embryo development, both *in vivo* and during assisted reproduction (13, 14).

Excessive ROS accumulation induces oxidative damage to lipids, proteins, and nucleic acids, including DNA strand breaks that may compromise genomic integrity in oocytes (15, 16). In particular, ROS-induced DNA damage — including single- and double-strand breaks — represents a major threat to genomic integrity in oocytes, which possess limited DNA repair capacity (15, 17). Because mitochondrial DNA lacks protective histones and efficient repair mechanisms, it is particularly susceptible to ROS-induced dysfunction affecting ATP production and chromosomal segregation during oocyte maturation (10, 14, 18).

The balance between ROS production and detoxification is maintained by an integrated antioxidant defense network comprising enzymatic and non-enzymatic antioxidants (19, 20). Collectively, these observations indicate that the follicular redox environment should be interpreted as a functionally interconnected system rather than as isolated biochemical markers (19–21). Such coordinated interactions raise the possibility that oxidative processes within follicular fluid may organize into biologically distinct and potentially opposing biochemical response patterns.

Increased body mass index (BMI) is associated with endocrine, inflammatory, and oxidative disturbances that may adversely affect female reproductive function (22). Obesity-related metabolic dysregulation and chronic low-grade inflammation promote oxidative imbalance and may disrupt the coordinated interactions within the follicular microenvironment (22–25). These findings suggest that obesity-related reproductive dysfunction may involve system-level reorganization of the follicular redox environment rather than isolated biochemical abnormalities.

In addition to endocrine alterations, obesity is accompanied by a persistent pro-inflammatory state that contributes to systemic metabolic dysregulation. Obesity is accompanied by chronic low-grade inflammation and enhanced oxidative stress (23, 26).This inflammatory background is closely intertwined with enhanced ROS production and reduced antioxidant capacity, resulting in a sustained shift in redox balance (27, 28). Importantly, these systemic perturbations are reflected at the level of the follicular microenvironment, where they may disrupt the finely coordinated interactions between oxidative and antioxidative pathways. These findings indicate that obesity-related metabolic and inflammatory signals affect reproductive outcomes not only through direct endocrine mechanisms, but also by reorganizing the redox landscape of the follicular environment — an effect that is unlikely to be captured by isolated biochemical parameters and that underscores the need for system-level analytical approaches.

Despite increasing evidence supporting the role of oxidative balance in follicular physiology, most previous studies have focused on isolated biomarkers or simple pairwise associations rather than on the coordinated organization of the follicular redox system (19, 29–32). Consequently, the existence of distinct oxidative phenotypes and coordinated directional response patterns within FF remains insufficiently understood.

In particular, there is a lack of studies investigating the directional consistency of associations between infertility-related factors and oxidative biomarkers, as well as the potential existence of distinct biochemical phenotypes within follicular fluid. Moreover, the influence of multiple, coexisting clinical and metabolic determinants — such as BMI and idiopathic infertility — on the organization of the redox system has not been systematically explored. Large population-based and cohort analyses have demonstrated significant associations between obesity-related parameters and infertility risk, yet these studies typically rely on conventional regression approaches and do not address system-level interactions between metabolic and oxidative processes (23, 33, 34).

Furthermore, although chronic inflammation and oxidative stress have been consistently implicated in impaired folliculogenesis, oocyte quality, and reproductive outcomes, existing evidence is largely derived from observational and mechanistic studies that do not integrate multidimensional biomarker interactions (8, 27, 29, 32, 35). Similarly, recent data indicate that metabolic and inflammatory factors jointly influence oxidative balance and fertility outcomes, yet their combined effects remain insufficiently characterized at the level of the follicular microenvironment (36–38).

Conventional statistical approaches based on independent outcome analyses may fail to capture latent structural patterns within such complex biological systems (39–41). Integrative analytical frameworks combining probabilistic modeling with unsupervised pattern recognition remain largely absent in studies of the follicular redox environment.

These limitations highlight the need for approaches capable of identifying coordinated patterns, directional effects, and latent structures within the follicular redox system. We hypothesized that oxidative and antioxidative biomarkers in follicular fluid are organized into distinct system-level phenotypes rather than acting as independent markers, and that the architecture of these phenotypes is differentially associated with clinical determinants of infertility, particularly BMI and idiopathic infertility. Such an approach may provide a more comprehensive understanding of the biological mechanisms underlying female infertility beyond the resolution offered by isolated biomarker analyses.

## 2 Materials and methods

This prospective observational (cross-sectional) study had two primary objectives. First, to assess the directional associations between infertility-related clinical and metabolic factors and oxidative biomarkers in human follicular fluid. Second, to investigate whether these associations reveal coordinated and potentially polarized patterns within the follicular redox system. A total of 77 women undergoing IVF treatment for infertility were enrolled in the study. All participants were recruited at TFP Fertility, Vitrolive, Poland. The characteristics of the study population are presented in **Table 1**. Ovarian stimulation was performed using gonadotropin preparations within either antagonist or agonist protocols. In the antagonist protocol, ganirelix (0.25 mg/day) was administered to prevent a premature luteinizing hormone surge. In the agonist protocol, triptorelin was initiated on day 20 of the preceding menstrual cycle to achieve pituitary downregulation. Ovarian stimulation was carried out using recombinant follitropin alfa or menotropin, with starting doses individualized according to ovarian reserve markers, particularly anti-Müllerian hormone (AMH) levels. The administered gonadotropin doses ranged from 150 to 375 IU per day, depending on the patient’s ovarian reserve and clinical response. Both stimulation protocols are routinely used in clinical IVF practice and were selected according to individual clinical indications. Final oocyte maturation was triggered with either recombinant human chorionic gonadotropin (two ampoules of 6,500 IU each) or triptorelin 0.2 mg.

**Table 1:** Descriptive statistics of infertility risk factors and oxidative enzyme concentration in follicular fluid, including sample size and missing data, among study group.

Risk factor/clinical response	Status/value	n	%	NAs	Missing
ovarian factor	no/yes	57/18	76/24	2	2.6%
tubal factor	no/yes	64/11	85.3/14.7	2	2.6%
idiopathic factor	no/yes	53/22	70.7/29.3	2	2.6%
endometriosis	no/yes	69/5	93.2/6.8	3	3.9%
male factor	no/yes	38/39	49.4/50.6	0	0%
IVF attempt number	1	54	70.1	0	0.0%
	2	11	14.3		
	3	6	7.8		
	4	3	3.9		
	5	2	2.6		
	6	1	1.3		
Risk factor/clinical response	n	Mean ± SD, median	Range	NAs	Missing
age	77	33.3 ± 4.3, 34.0	23.0-44.0	0	0.0%
number of hMG/FSH administration days	77	9.6 ± 2.0, 9.0	4.0-14.0	0	0.0%
AMH (anti-Müllerian hormone) [ng/mL]	76	3.47 ± 2.93, 2.72	0.40-16.81	1	1.3%
BMI (body mass index) Protein (R) [kg/m^2^]	74	22.7 ± 3.5, 21.8	17.4-36.3	3	3.9%
enzyme	n	Mean ± SD, Median	Range	NAs	Missing
Protein (R) [g/L]	70	51.24 ± 7.49, 49.94	37.44–75.24	7	9.1%
Protein (L) [g/L]	71	51.80 ± 8.08, 50.74	36.59–71.85	6	7.8%
SH (R) [µmol/L]	70	302.08 ± 84.12, 278.69	129.23–543.50	7	9.1%
SH (L) [µmol/L]	71	307.47 ± 92.52, 285.53	157.22–585.18	6	7.8%
SH/Protein (R) [µmol/g protein]	70	6.02 ± 1.97, 5.31	3.10–10.92	7	9.1%
SH/Protein (L) [µmol/g protein]	71	6.06 ± 2.02, 5.44	3.38–11.94	6	7.8%
CER (R) [mg/dL]	70	26.87 ± 9.78, 26.68	9.73–58.70	7	9.1%
CER (L) [mg/dL]	71	26.71 ± 8.73, 25.29	11.73–60.91	6	7.8%
TAC (R) [mmol/L]	70	0.87 ± 0.12, 0.86	0.43–1.22	7	9.1%
TAC (L) [mmol/L]	71	0.88 ± 0.13, 0.88	0.35–1.16	6	7.8%
TOS (R) [µmol/L]	70	7.99 ± 2.92, 8.48	0.00–14.47	7	9.1%
TOS (L) [µmol/L]	71	7.75 ± 2.84, 8	1.76–16.33	6	7.8%
LF (R) (RF)	70	291 ± 109, 3	0.00–5.35	7	9.1%
LF (L) (RF)	71	283 ± 107, 3	0.00–6.04	6	7.8%
GR (R) [IU/L]	70	24.96 ± 6.09, 25.63	0.58–36.36	7	9.1%
GR (L) [IU/L]	71	26.21 ± 7.44, 24.95	13.81–55.40	6	7.8%
GR/Protein (R) [IU/L protein]	70	0.49 ± 0.13, 0.52	0.01–0.72	7	9.1%
GR/Protein (L) [IU/L protein]	71	0.51 ± 0.15, 0.5	0.16–0.99	6	7.8%
CAT (R) [IU/L]	70	8.07 ± 3.75, 7.36	3.02–21.29	7	9.1%
CAT (L) [IU/L]	71	8.44 ± 4.39, 7.16	2.54–31.97	6	7.8%
CAT/Protein (R) [IU/g protein]	70	0.16 ± 0.07, 0.14	0.05–0.44	7	9.1%
CAT/Protein (L) [IU/g protein]	71	0.17 ± 0.11, 0.14	0.06–0.82	6	7.8%
SOD (R) [NU/mL]	70	17.90 ± 3.67, 17.78	9.92–24.71	7	9.1%
SOD (L) [NU/mL]	71	18.69 ± 4.63, 19.28	8.95–27.90	6	7.8%
MnSOD (R) [NU/mL]	70	8.81 ± 1.85, 8.62	5.07–13.87	7	9.1%
MnSOD (L) [NU/mL]	71	9.00 ± 1.75, 9.08	4.11–13.60	6	7.8%
CuZnSOD (R) [NU/mL]	70	9.09 ± 2.88, 9.11	3.01–15.71	7	9.1%
CuZnSOD (L) [NU/mL	71	9.70 ± 4.04, 9.74	2.68–19.74	6	7.8%
GPX (R) [IU/L]	70	974.26 ± 158.08, 943.12	585.47–1336.07	7	9.1%
GPX (L) [IU/L]	71	996.58 ± 192.52, 974.05	462.64–1398.99	6	7.8%
GPX/Protein (R) [IU/g protein]	70	19.27 ± 3.47, 19.25	11.27–27.35	7	9.1%
GPX/Protein (L) [IU/g protein]	71	19.58 ± 4.50, 19.06	12.03–33.41	6	7.8%
GST (R) [IU/L]	70	7.34 ± 2.38, 7.21	0.00–14.61	7	9.1%
GST (L) [IU/L]	71	7.39 ± 2.28, 6.9	3.16–13.42	6	7.8%
GST/Protein (R) [IU/g protein]	70	0.15 ± 0.05, 0.14	0.00–0.31	7	9.1%
GST/Protein (L) [IU/g protein]	71	0.14 ± 0.05, 0.14	0.07–0.35	6	7.8%
MDA (R) [IU/g protein]	70	0.26 ± 0.13, 0.23	0.05–0.60	7	9.1%
MDA (L) [IU/g protein]	71	0.24 ± 0.10, 0.23	0.08–0.51	6	7.8%

SH, sulfhydryl groups; CER, ceruloplasmin; TAC, total antioxidant capacity; TOS, total oxidant status;LF , lipofuscin; CAT, catalase; GR, glutathione reductase; GPX, glutathione peroxidase; GST, glutathione S-transferase; SOD, superoxide dismutase; MnSOD, manganese superoxide dismutase; CuZnSOD, copper-zinc superoxide dismutase; MDA, malondialdehyde.

Follicular fluid (FF) oxidative stress and antioxidant system markers were analyzed in samples obtained from 77 patients at the Department of Biochemistry, Silesian Medical University, School of Medicine, and the Division of Dentistry in Zabrze, Poland.

The study was approved by the Bioethics Committee of the Regional Medical Chamber in Szczecin, Poland (approval no. 12/KB/VI/2016). Written informed consent was obtained from all participants prior to inclusion in the study and sample collection.

### 2.1 Follicular fluid collection and processing

Follicular fluid was collected once from each participant during transvaginal oocyte retrieval performed 36 hours after ovulation triggering within a single IVF cycle. During the aspiration procedure, follicular fluid was obtained separately from the dominant follicles of the right and left ovary, yielding two samples per patient that were treated as paired ovarian measurements in subsequent analyses. No repeated sampling from subsequent IVF cycles was included in the study. To avoid contamination with blood, aspirates with visible haemorrhagic discolouration were excluded from analysis. FF samples were centrifuged at 10,000 × g for 10 minutes at 4°C, and supernatants were aliquoted and stored at −80°C until analysis. Total follicular fluid protein concentration was quantified using an A25 biochemistry analyzer (BioSystems S.A., Barcelona, Spain) according to the manufacturer’s protocol. Enzyme activities normalized to protein concentration are reported alongside absolute activities where applicable.

### 2.2 Biochemical analyses

Each follicular fluid sample was analyzed using a predefined panel of 19 redox-related parameters selected to characterize complementary components of the follicular oxidative–antioxidative system. The panel was designed to capture global oxidant and antioxidant status, thiol-dependent buffering capacity, superoxide scavenging, peroxide detoxification, glutathione recycling, glutathione-dependent detoxification, protein-adjusted enzymatic activity, and lipid peroxidation-related damage. The following parameters were measured: total protein, protein sulfhydryl (SH) groups, protein-bound sulfhydryl groups normalized to protein (SH/Protein), ceruloplasmin (CER), total antioxidant capacity (TAC), total oxidant status (TOS), lipofuscin (LF), total superoxide dismutase (SOD), and its isoenzymes manganese (MnSOD) and copper–zinc superoxide dismutase (CuZnSOD), glutathione peroxidase (GPX), GPX normalized to protein (GPX/Protein), glutathione reductase (GR), GR normalized to protein (GR/Protein), catalase (CAT), CAT normalized to protein (CAT/Protein), glutathione S-transferase (GST), GST normalized to protein (GST/Protein), and malondialdehyde (MDA). Total protein, SH, SH/Protein, CER, TAC, TOS, LF, and MDA were included to describe the general biochemical and oxidative status of follicular fluid, whereas SOD isoenzymes, GPX, CAT, GR, GST, and their selected protein-normalized ratios were included to reflect major enzymatic antioxidant and detoxification pathways relevant to follicular redox homeostasis. No predefined biomarker was excluded from the analysis because of partial non-availability of measurements; missing biochemical values were retained and handled within the Bayesian framework as latent variables.

#### 2.2.1 Antioxidant enzymes

Total SOD activity and its isoenzymes—manganese (MnSOD) and copper–zinc superoxide dismutase (CuZnSOD)—were determined using the nitrite method of Oyanagui (42), based on superoxide-mediated oxidation of hydroxylamine to nitrite, which was subsequently detected as a diazo dye complex at 550 nm. One nitrite unit (NU) was defined as the amount of enzyme causing 50% inhibition of nitrite formation. Isoenzyme activities were differentiated by parallel incubation with and without 1 mM potassium cyanide (KCN), a selective inhibitor of CuZnSOD. The residual activity measured in the presence of KCN was attributed to MnSOD, whereas CuZnSOD activity was calculated as the difference between total SOD and MnSOD activity (42).

Catalase (CAT) activity was assessed using the Johansson and Borg method (43). The assay measures formaldehyde produced from the reaction between methanol and H*_2_*O*_2_* in the presence of CAT; absorbance of the purpald–formaldehyde complex was recorded at 550 nm. Results were expressed as U/g protein.

Glutathione peroxidase (GPX) activity was measured using a kinetic spectrophotometric method based on the coupled reaction with glutathione reductase, NADPH, and hydrogen peroxide as the substrate (44). The decrease in absorbance at 340 nm, reflecting NADPH oxidation, was used to calculate enzyme activity. Results were expressed as IU/mL and as IU/g protein (GPX/Protein).

Glutathione reductase (GR) activity was determined spectrophotometrically by monitoring the oxidation of NADPH at 340 nm in the presence of oxidized glutathione (GSSG) as the substrate (45, 46). Results were expressed as IU/mL and as IU/g protein (GST/Protein).

Glutathione S-transferase (GST) activity was measured using 1-chloro-2,4-dinitrobenzene (CDNB) as the substrate, according to the method of Habig et al. (47) The rate of CDNB conjugation with reduced glutathione was monitored spectrophotometrically at 340 nm. Results were expressed as IU/mL and as IU/g protein (GST/Protein).

#### 2.2.2 Non-enzymatic antioxidants and oxidative stress markers

Total antioxidant capacity (TAC) was quantified according to Erel (48). This colorimetric assay measures the capacity of antioxidants in the follicular fluid to suppress the formation of ABTS^+^ radicals (2,2′-azinobis(3-ethylbenzothiazoline)-6-sulfonate). The decrease in absorbance at 660 nm indicates antioxidant activity. Analyses were performed using an automated PerkinElmer analyzer calibrated with Trolox (Sigma-Aldrich); results were expressed in mmol/L.

Ceruloplasmin (CER) concentration was assessed according to the method of Richterich (46), with absorbance recorded at 546 nm. Values were expressed in mg/dL.

Protein sulfhydryl (SH) groups were measured following the procedure of Koster et al. (49). In this assay, 5,5′-dithiobis (2-nitrobenzoic acid) (DTNB) is reduced by thiol-containing compounds, producing the yellow 5-thio-2-nitrobenzoate anion, which is detected at 412 nm. Measurements were performed using an automated PerkinElmer analyzer (USA). Results were expressed as µmol/g protein (SH/Protein) and as absolute concentrations.

Total oxidant status (TOS) was evaluated using Erel’s method (50), in which oxidants present in the follicular fluid sample oxidize ferrous ions into ferric ions in an acidic medium. The ferric–xylenol orange complex was quantified at 560 nm on a PerkinElmer analyzer. TOS results were expressed in μmol/L.

Lipofuscin (LF) was quantified according to Jain (51). Lipofuscin fluorescence was measured fluorometrically and expressed in relative fluorescence units (RF) corresponding to a fluorescence standard of 0.1 mg/mL quinidine sulfate in 0.1N sulfuric acid. In the context of follicular fluid, LF serves as a marker of cumulative oxidative damage to cellular macromolecules, reflecting long-term oxidative burden within the follicular microenvironment.

Malondialdehyde (MDA) was measured using the thiobarbituric acid-reactive substances (TBARS) method of Ohkawa et al. (52). Samples were incubated with sodium dodecyl sulphate, acetic acid, and thiobarbituric acid at 95°C for 1 hour, extracted with butanol–pyridine, and the organic phase was read fluorometrically at 552 nm (excitation 515 nm) using a SHIMADZU fluorometer. Results were expressed as MDA equivalents using tetraethoxypropane as the standard (μmol/L).

### 2.3 Statistical analysis

Statistical analysis was performed in three sequential stages: (i) Bayesian multivariable regression to quantify directional associations between clinical risk factors and individual biochemical markers; (ii) taxonomic clustering of markers based on the resulting directional association patterns; and (iii) comparison of cluster-specific directional distributions using Fisher’s exact test. The analytical framework was specifically designed to identify coordinated directional patterns and latent polarization structures within the follicular redox system.

#### 2.3.1 Bayesian multivariable regression and risk factor selection

Initially, the analysis included ten candidate explanatory variables and 19 oxidative biomarker responses measured in follicular fluid from both the right and left ovary in 77 patients, with two paired ovarian measurements per patient (**Table 1**). To account for potential clinical confounding at the model-building stage, the candidate predictor set included variables reflecting age-related effects, ovarian reserve, infertility diagnosis, stimulation exposure, and metabolic status. Specifically, age, AMH concentration, duration of hMG/FSH stimulation, IVF attempt number, ovarian factor, tubal factor, male factor, idiopathic infertility, endometriosis, and BMI were initially considered in the Bayesian multivariable framework. Thus, age, ovarian reserve status, infertility etiology, and stimulation-related exposure were evaluated before the final set of retained predictors was established. Due to missing (non-available = NA) values in the dataset of risk factors and oxidative biomarker concentrations measured in follicular fluid from both the right and left ovary, statistical analyses were performed within a Bayesian framework using the WinBUGS computational platform (53).

In this approach, missing observations were treated as latent random variables and estimated jointly with the remaining model parameters. This enabled the use of all available data without excluding incomplete records while appropriately accounting for uncertainty associated with missingness.

Unlike conventional single-imputation methods, which replace missing values with fixed estimates, WinBUGS applies a data augmentation procedure. During each iteration of the Markov chain Monte Carlo (MCMC) algorithm, missing values were sampled from their conditional posterior distributions given the observed data and the specified model. This approach preserves the full statistical model structure, avoids underestimation of variability, and yields credible intervals that incorporate imputation uncertainty, thereby ensuring coherent statistical inference.

For each of the 19 biochemical outcomes (see **Table 1**), a bivariate linear regression model with fixed effects was fitted to jointly analyse paired ovarian measurements while accounting for within-patient dependence:


$${\text{y}}_{\text{i}}={\left({\text{y}}_{\text{iR}},{\text{y}}_{\text{iL}}\right)}^{⊤}$$

$${\text{y}}_{\text{i}}=\text{a}+\sum_{\text{j=}1}^{\text{k}}{\text{β}}_{\text{j}}{\text{x}}_{\text{ij}}+{\text{ϵ}}_{\text{i}}$$

where y_i_ denotes the response vector for the i-th patient, corresponding to measurements from the right (y_iR_) and left (y_iL_) ovary; a is the intercept vector; β_j_ are regression coefficient vectors for the j-th explanatory variable; and ϵ_i_ is a residual error term assumed to follow a bivariate normal distribution with an unstructured covariance matrix.

Diffuse prior distributions were assigned to model parameters, with regression coefficients specified as: a, β_i_∼N(0,1.0×10^−6^) and the precision parameter as: τ∼Gamma(1.0×10^−3^,1.0×10^−3^). Posterior inference was based on Markov chain Monte Carlo (MCMC) simulations using multiple chains with overdispersed initial values. Convergence was assessed using standard diagnostic procedures, including visual inspection of trace plots and the Gelman–Rubin statistic (53). Results are presented as posterior means with 95% credible intervals (CrI). A regression coefficient was considered statistically (posteriorly) significant if its 95% CrI did not include zero, which approximately corresponds to a two-sided significance level of α ≈ 0.05.

Initially, all ten candidate explanatory variables (**Table 1**) were included in multivariable regression models for each of the 19 oxidative enzyme outcomes; following backward elimination based on the frequency of significant Boolean posterior indicators across outcomes, the model was refitted retaining the four variables with the strongest and most consistent credible associations: ovarian factor, male factor, idiopathic factor, and BMI.

For each regression coefficient, posterior directional probabilities were derived from MCMC simulations and used to assess both the stability and direction of effects. Associations were classified based on Boolean posterior node criteria: values <0.025 indicated a statistically significant positive (direct) association, coded as “1”, whereas values >0.975 indicated a statistically significant negative (inverse) association, coded as “−1”. Posterior probabilities between 0.025 and 0.975 were considered non-significant and coded as “0”.

#### 2.3.2 Taxonomic clustering of biochemical phenotypes

Subsequently, based on the values of the Boolean indicators determining the directions of the effects of risk factors on the clinical responses, pairwise dissimilarities among the 19 oxidative enzyme concentrations measured in follicular fluid were quantified using the Marczewski–Steinhaus (M–S) metric of taxonomic differences (54). In classical applications, variables entered into the M–S metric are first normalized to the [0,1] interval using the transformation:


$${\text{x}}_{\text{n}}=({\text{x}}_{\text{i}}-{\text{x}}_{\text{min}})/({\text{x}}_{\text{max}}-{\text{x}}_{\text{min}})$$

ensuring comparability across variables measured on different scales.

In the present study, however, instead of normalized raw biochemical values, posterior Boolean indicator values derived from the Bayesian regression models were used to construct the distance matrix. These indicators also naturally lie within the [0,1] range, as they represent posterior probabilities describing the direction and stability of regression coefficients. Thus, rather than measuring taxonomic distances between patients, the M–S framework was repurposed to evaluate similarities and dissimilarities among enzymes according to their multivariable association patterns with infertility risk factors.

For two compared objects (enzymes) A and B, the symmetric taxonomic distance was defined as:


D=∣A−B∣/max(A,B)

where the numerator represents the absolute difference and the denominator the larger of the two values.

This metric reflects relative rather than absolute differences, making it particularly suitable for identifying structural similarity patterns among biomarkers. In contrast to regression-based approaches, which assess predefined predictor–outcome relationships, the M–S framework is unsupervised and characterizes the intrinsic geometric organization of the data without imposing linear assumptions.

The resulting distance matrix was then used to generate a taxonomic dendrogram, enabling classification of the 19 follicular fluid enzymes into major branches of clinical phenotypes with similar statistical characteristics using the AGNES (Agglomerative Nesting) hierarchical clustering method (55).

#### 2.3.3 Inter-phenotype comparison

In the final stage, the distributions of directional effect codes (−1, 0, 1), representing inverse, non-significant, and direct associations, respectively, were compared between Phenotype 1 and Phenotype 2, corresponding to the two enzyme branches identified by taxonomic classification. For each of the four selected risk factors, contingency tables were constructed and analyzed using Fisher’s exact test. This procedure was used to determine whether the pattern of statistically significant associations differed between the two major biochemical phenotype clusters. Calculations were performed in the R statistical environment (56, 57).

## 3 Results

### 3.1 Missing data

Missingness among clinical risk factors was low, ranging from 0.0% to 3.9% (**Table 1**). All 19 biochemical markers showed an identical pattern of missingness, with 13 missing observations each (8.4%), consistent with unavailable paired samples rather than marker-specific measurement failure. Missing values were handled within the Bayesian framework as described in Section 2.3.1.

### 3.2 Risk factor selection and Bayesian regression results

Following Bayesian variable selection, four factors — ovarian factor, male factor, idiopathic infertility, and BMI — were retained in the final multivariable model due to the highest frequency of significant directional associations across oxidative biomarkers (**Table 2**). These four variables yielded the greatest number of statistically significant posterior associations across the 19 biochemical outcomes. The posterior probabilities and directional association codes (−1, 0, 1) for each biomarker–risk factor pair, derived from the four-factor MCMC model, are presented in **Table 2** (Panel A), together with the phenotype assignment resulting from the taxonomic clustering step (Panel B). Notably, directional association patterns already suggested the presence of two opposing oxidative response architectures, later confirmed by taxonomic clustering analysis.

**Table 2:** Panel A: posterior probabilities and directional association codes for selected risk factors and follicular fluid biochemical markers.

Panel:	A				B
Enzyme	Ovarian factor	Male factor	Idiopathic factor	BMI	Phenotype
Protein	0.7556 (0)	0.9808 (-1)	0.9847 (-1)	0.9911 (-1)	1
SH	0.8645 (0)	0.0002 (1)	0.8193 (0)	0.9353 (0)	1
SH/Protein	0.7833 (0)	0.0000 (1)	0.5371 (0)	0.7254 (0)	1
CER	0.0062 (1)	0.0043 (1)	0.9482 (0)	0.5188 (0)	1
TAC	0.8361 (0)	0.0103 (1)	0.9986 (-1)	0.9463 (0)	1
TOS	0.9957 (-1)	0.0635 (0)	0.9964 (-1)	0.9883 (-1)	1
LF	0.9958 (-1)	0.0697 (0)	0.9969 (-1)	0.9833 (-1)	1
GR	0.0857 (0)	0.2686 (0)	0.1110 (0)	0.3213 (0)	2
GR/Protein	0.0208 (1)	0.0576 (0)	0.0084 (1)	0.0548 (0)	2
CAT	0.2129 (0)	0.6724 (0)	0.0154 (1)	0.0124 (1)	2
CAT/Protein	0.1325 (0)	0.4551 (0)	0.0039 (1)	0.0022 (1)	2
SOD	0.9897 (-1)	0.6731 (0)	0.8498 (0)	0.9298 (0)	1
MnSOD	0.9823 (-1)	0.7466 (0)	0.9935 (-1)	0.9994 (-1)	1
CuZnSOD	0.9620 (0)	0.5685 (0)	0.5005 (0)	0.5243 (0)	1
GPX	0.6865 (0)	0.0297 (0)	0.9995 (-1)	0.9955 (-1)	1
GPX/Protein	0.5317 (0)	0.0024 (1)	0.9480 (0)	0.7845 (0)	1
GST	0.9012 (0)	0.9976 (-1)	0.1617 (0)	0.0855 (0)	2
GST/Protein	0.8516 (0)	0.9734 (0)	0.0351 (0)	0.0140 (1)	2
MDA	0.7098 (0)	0.1663 (0)	0.8200 (0)	0.9710 (0)	1

Values <0.025 or >0.975 were considered posteriorly significant and indicate inverse (−1) or direct (+1) associations, respectively, whereas intermediate values were interpreted as non-significant (0). Panel B: Taxonomic phenotype assignment.

### 3.3 Taxonomic distance matrix and dendrogram

**Figure 1:**
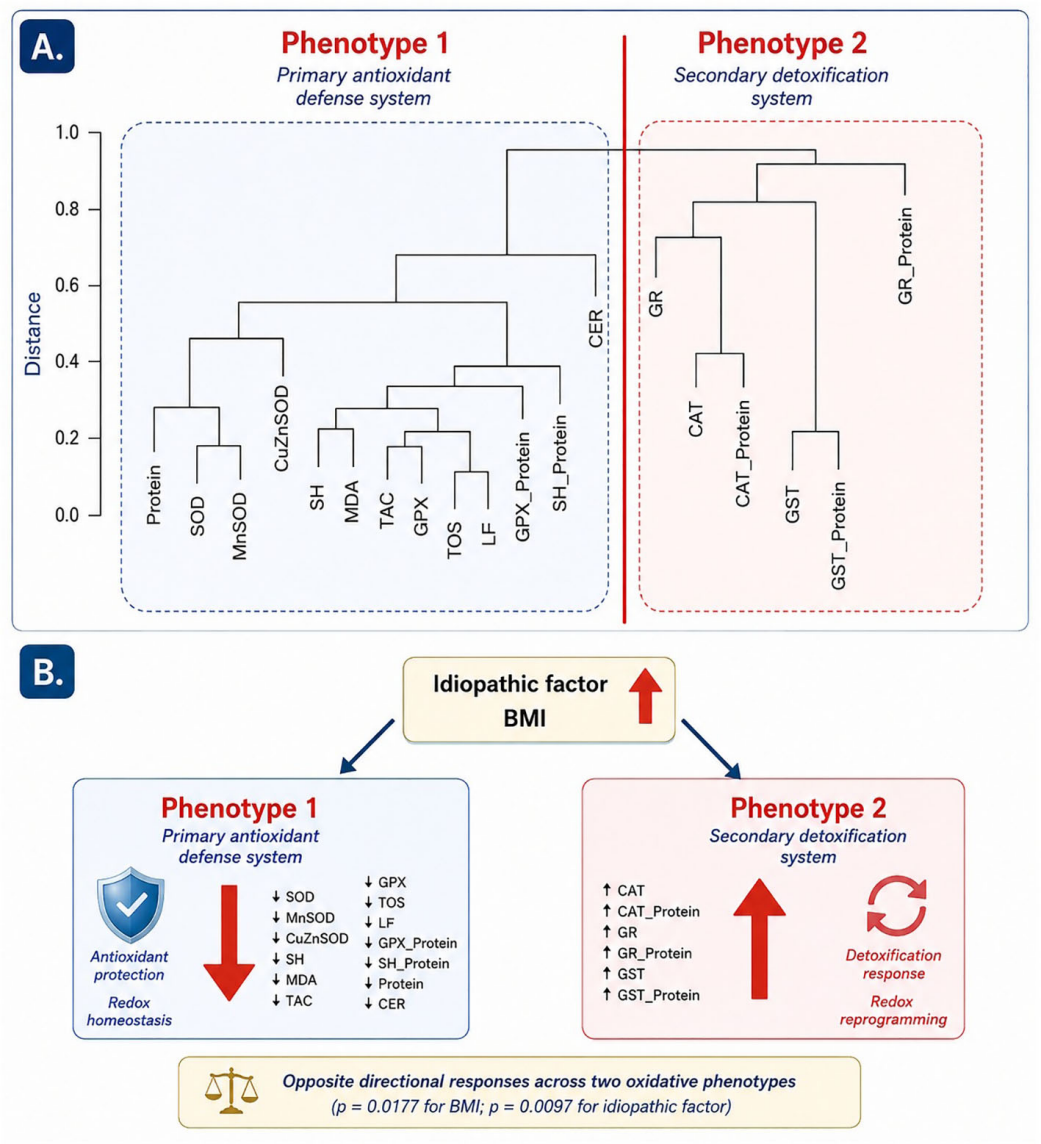
Unsupervised taxonomic classification of oxidative biomarkers based on Bayesian directional association patterns. Panel **(A)** presents the AGNES hierarchical clustering dendrogram constructed from the Marczewski–Steinhaus distance matrix derived from Boolean posterior directional indicators. Two major branches were identified and designated Phenotype 1 and Phenotype 2. Panel **(B)** summarizes the predominant directional effects of BMI and idiopathic infertility on biomarkers assigned to each phenotype.

The taxonomic distance matrix for the 19 oxidative enzymes, constructed from posterior probabilities of Boolean posterior indicators using the Marczewski–Steinhaus metric, is presented in **Table 3**. Based on this matrix, agglomerative hierarchical clustering (AGNES algorithm) yielded the dendrogram presented in **Figure 1A**. The dendrogram provides a graphical representation of the taxonomic relationships among the 19 oxidative biomarkers based on the similarity of their directional association patterns with the four selected infertility risk factors. Biomarkers connected at lower linkage heights exhibit more similar posterior directional profiles, whereas biomarkers joined at greater heights display increasingly divergent response patterns. The highest linkage level separating the two main branches reflects the maximal taxonomic dissimilarity observed in the dataset and supports the distinction of two major oxidative phenotypes.

**Table 3:** Taxonomic distance matrix for the 19 oxidative enzymes, constructed using the Marczewski–Steinhaus metric.

Enzyme	Protein	SH	SH/protein	CER	TAC	TOS	LF	GR	GR/protein	CAT	CAT/protein	SOD	MnSOD	CuZnSOD	GPX	GPX/protein	GST	GST/protein	MDA
Protein	0	0.34	0.46	0.6	0.29	0.3	0.3	0.79	0.96	0.75	0.84	0.19	0.12	0.4	0.28	0.39	0.49	0.53	0.28
SH	0.34	0	0.22	0.51	0.08	0.14	0.14	0.82	0.97	0.93	0.95	0.24	0.3	0.42	0.16	0.22	0.7	0.75	0.13
SH/Protein	0.46	0.22	0	0.57	0.27	0.33	0.33	0.78	0.96	0.91	0.94	0.41	0.45	0.35	0.31	0.29	0.67	0.73	0.28
CER	0.6	0.51	0.57	0	0.47	0.51	0.51	0.76	0.95	0.98	0.99	0.61	0.6	0.66	0.46	0.35	0.92	0.98	0.52
TAC	0.29	0.08	0.27	0.47	0	0.08	0.08	0.83	0.97	0.93	0.95	0.27	0.25	0.46	0.08	0.19	0.72	0.76	0.16
TOS	0.3	0.14	0.33	0.51	0.08	0	0	0.82	0.95	0.92	0.94	0.22	0.19	0.42	0.12	0.26	0.7	0.76	0.19
LF	0.3	0.14	0.33	0.51	0.08	0	0	0.82	0.95	0.91	0.94	0.22	0.19	0.42	0.12	0.26	0.69	0.75	0.18
GR	0.79	0.82	0.78	0.76	0.83	0.82	0.82	0	0.82	0.71	0.65	0.77	0.79	0.69	0.81	0.79	0.77	0.82	0.75
GR/Protein	0.96	0.97	0.96	0.95	0.97	0.95	0.95	0.82	0	0.9	0.87	0.96	0.96	0.94	0.96	0.96	0.93	0.95	0.95
CAT	0.75	0.93	0.91	0.98	0.93	0.92	0.91	0.71	0.9	0	0.35	0.73	0.75	0.7	0.92	0.92	0.57	0.51	0.87
CAT/Protein	0.84	0.95	0.94	0.99	0.95	0.94	0.94	0.65	0.87	0.35	0	0.83	0.84	0.77	0.95	0.95	0.72	0.68	0.9
SOD	0.19	0.24	0.41	0.61	0.27	0.22	0.22	0.77	0.96	0.73	0.83	0	0.08	0.26	0.32	0.39	0.52	0.58	0.25
MnSOD	0.12	0.3	0.45	0.6	0.25	0.19	0.19	0.79	0.96	0.75	0.84	0.08	0	0.31	0.27	0.39	0.52	0.58	0.28
CuZnSOD	0.4	0.42	0.35	0.66	0.46	0.42	0.42	0.69	0.94	0.7	0.77	0.26	0.31	0	0.51	0.52	0.42	0.5	0.43
GPX	0.28	0.16	0.31	0.46	0.08	0.12	0.12	0.81	0.96	0.92	0.95	0.32	0.27	0.51	0	0.16	0.75	0.8	0.13
GPX/Protein	0.39	0.22	0.29	0.35	0.19	0.26	0.26	0.79	0.96	0.92	0.95	0.39	0.39	0.52	0.16	0	0.78	0.84	0.23
GST	0.49	0.7	0.67	0.92	0.72	0.7	0.69	0.77	0.93	0.57	0.72	0.52	0.52	0.42	0.75	0.78	0	0.13	0.7
GST/Protein	0.53	0.75	0.73	0.98	0.76	0.76	0.75	0.82	0.95	0.51	0.68	0.58	0.58	0.5	0.8	0.84	0.13	0	0.74
MDA	0.28	0.13	0.28	0.52	0.16	0.19	0.18	0.75	0.95	0.87	0.9	0.25	0.28	0.43	0.13	0.23	0.7	0.74	0

In this scale, a value of 0 denotes the minimum taxonomic distance (no difference), whereas a value of 1 denotes the maximum taxonomic difference. The matrix is symmetric, and all diagonal elements are equal to 0, reflecting that the distance between identical enzymes is necessarily zero.

### 3.4 Two polarized biochemical phenotypes

Hierarchical clustering identified two biologically distinct and antagonistically organized oxidative phenotypes. Phenotype 1 comprised 13 biomarkers: Protein, SOD, MnSOD, CuZnSOD, SH, MDA, TAC, GPX, TOS, LF, GPX/Protein, SH/Protein, and CER. Phenotype 2 comprised six biomarkers: GR, CAT, CAT/Protein, GST, GST/Protein, and GR/Protein. The large taxonomic distance separating the two clusters indicates fundamentally different directional association patterns between the identified phenotypes. Phenotype assignments are presented in **Table 2**, Panel B.

### 3.5 Inter-phenotype comparison of directional associations

The distributions of directional association codes were compared between Phenotype 1 and Phenotype 2 for each of the four retained risk factors using Fisher’s exact test (**Table 4**). Statistically significant differences in the pattern of directional associations between phenotypes were observed for idiopathic factor infertility (p = 0.0097) and BMI (p = 0.0177). No significant inter-phenotype differences were found for ovarian factor (p = 0.3148) or male factor (p = 0.2459). In both significant comparisons, Phenotype 1 was characterized predominantly by inverse associations (coded −1), whereas Phenotype 2 showed predominantly direct associations (coded 1).

**Table 4:** Distribution of directional association codes across oxidative enzyme phenotypes and results of Fisher’s exact test.

Risk factor	Phenotype	Inverse association (−1)	Non-significant (0)	Direct association (1)	p-value
Ovarian factor	1	4	8	1	0.3148
	2	0	5	1	
Male factor	1	1	7	5	0.2459
	2	1	5	0	
Idiopathic factor	1	6	7	0	0.0097
	2	0	3	3	
BMI	1	5	8	0	0.0177
	2	0	3	3	

The integrated analytical framework consistently identified idiopathic infertility and BMI as the principal drivers of oxidative phenotype polarization within follicular fluid. Both factors induced opposite directional shifts across the two identified biochemical phenotypes, revealing an antagonistically organized redox response architecture not observed for other infertility factors (**Figure 1B**).

### 3.6 Graphical summary of the analytical workflow and main findings

**Figure 2:**
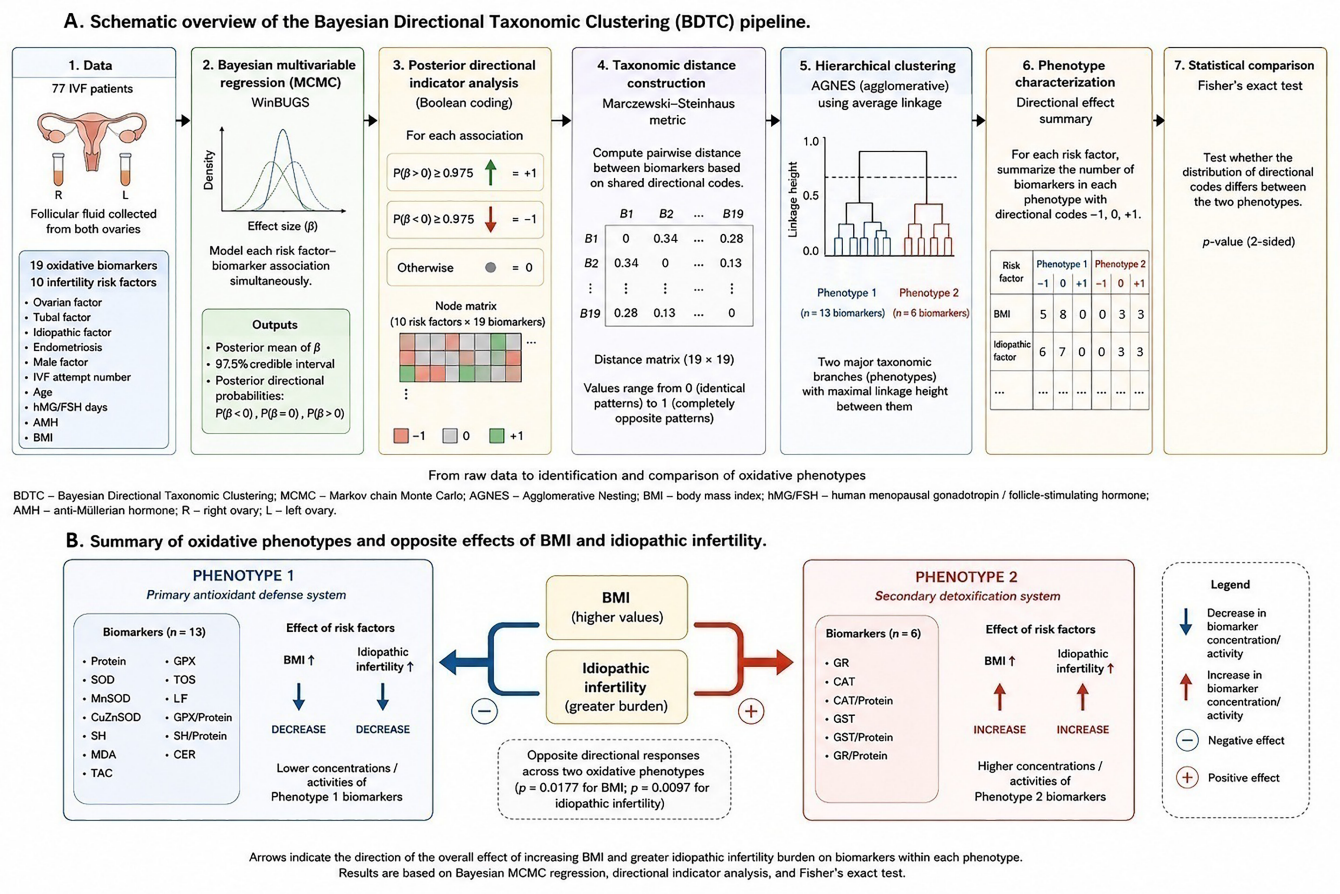
Graphical summary of the Bayesian Directional Taxonomic Clustering pipeline and oxidative phenotype polarization. **(A)** shows the analytical workflow from paired follicular fluid data to phenotype identification and statistical comparison. **(B)** summarizes the opposite effects of increasing BMI and idiopathic infertility burden on the two identified oxidative phenotypes.

To provide an integrated overview of the analytical strategy and its biological interpretation, the complete Bayesian Directional Taxonomic Clustering (BDTC) pipeline is summarized in **Figure 2**. Panel A presents the sequential workflow of the study, including data acquisition from paired follicular fluid samples, Bayesian multivariable regression, posterior directional indicator analysis, construction of the Marczewski–Steinhaus taxonomic distance matrix, hierarchical clustering, phenotype characterization, and inter-phenotype statistical comparison. Panel B summarizes the principal biological finding: increasing BMI and greater idiopathic infertility burden were associated with opposite directional responses across the two identified oxidative phenotypes, with decreased activity/concentration of Phenotype 1 biomarkers and increased activity/concentration of Phenotype 2 biomarkers.

## 4 Discussion

### 4.1 Identification of two polarized redox phenotypes: a system-level finding

The principal finding of the present study is the identification of two taxonomically distinct enzymatic phenotypes within follicular fluid (FF), designated Phenotype 1 and Phenotype 2, which exhibit antagonistic patterns of directional association with clinical infertility-related factors. This observation suggests that the redox microenvironment of the follicle is better understood as a structurally organized system rather than as a collection of isolated biomarker signals, with coordinated, system-level regulation shaping the enzymatic landscape surrounding the developing oocyte. The applied methodology — combining Bayesian Markov chain Monte Carlo (MCMC) regression, Boolean directional indicator analysis, and unsupervised Marczewski–Steinhaus taxonomic clustering — revealed this latent structure, which would remain undetected by conventional single-parameter or pairwise correlation analyses.

Current literature remains largely focused on isolated biomarker analyses. Total antioxidant capacity (TAC), superoxide dismutase (SOD), glutathione peroxidase (GPX), and catalase (CAT) are most commonly evaluated as independent parameters in the context of assisted reproductive technology (ART) outcomes, without addressing their coordinated interrelationships (5, 29, 58–60). However, accumulating evidence indicates that follicular fluid contains a complex mixture of steroids, metabolites, reactive oxygen species, and antioxidant enzymes whose mutual interactions — rather than individual concentrations — are likely to determine follicular function and oocyte developmental competence (58, 61). The present results extend this concept by demonstrating that FF enzymes cluster into two qualitatively distinct branches based on the directionality of their clinical associations, supporting the notion that redox regulation in the follicular microenvironment operates through a structured, polarized mechanism rather than through uniform up- or downregulation.

From a biological perspective, the two phenotypes may represent different functional states of the follicular antioxidant network. The attenuation of Phenotype 1 biomarkers, including MnSOD, GPX, TAC, and thiol-related markers, may indicate reduced primary antioxidant buffering capacity within the follicular microenvironment. Such a pattern could expose the oocyte–cumulus complex to increased mitochondrial ROS burden, impaired peroxide clearance, and oxidative damage affecting mitochondrial DNA, proteins, lipids, and cellular signaling. In contrast, the increase in Phenotype 2 enzymes, including CAT, GR, and GST, may represent a secondary or compensatory detoxification response activated under persistent metabolic or inflammatory redox stress. Thus, the observed polarization may not simply reflect “more” or “less” oxidative stress, but rather a redistribution of antioxidant defense from primary redox buffering toward downstream detoxification pathways.

This interpretation is biologically relevant to oocyte competence and ART outcomes, although it requires prospective validation. Mitochondrial function within the oocyte–cumulus complex is essential for ATP production, meiotic spindle integrity, chromosomal segregation, cytoplasmic maturation, fertilization, and early embryo development. Previous studies have shown that mitochondrial dysfunction in cumulus cells is associated with reduced mtDNA copy number, lower ATP production, down-regulation of oxidative phosphorylation-related genes, and decreased reproductive capacity (14). In this context, Phenotype 1 suppression may reflect a follicular environment less capable of maintaining mitochondrial redox homeostasis, whereas Phenotype 2 activation may indicate increased demand for detoxification under sustained oxidative pressure. Therefore, the identified phenotypes may provide a mechanistic link between BMI, idiopathic infertility, altered follicular redox organization, and impaired oocyte developmental potential. However, because embryological and reproductive outcomes were not included in the present analysis, the prognostic value of these phenotypes for IVF outcomes should be tested in larger prospective cohorts.

From a methodological standpoint, the use of Bayesian data augmentation for handling missing observations as latent random variables — rather than applying single-imputation or listwise deletion — preserves the full statistical model structure and yields credible intervals incorporating imputation uncertainty. This approach ensures coherent inference in the presence of the modest missingness (8.4%) observed among biochemical outcomes, which likely reflected common technical constraints rather than marker-specific measurement failure.

### 4.2 Phenotype 1: suppression of primary antioxidant defenses and compartment-specific behavior of oxidative stress markers

Phenotype 1 comprises 13 markers — including MnSOD, SOD, CuZnSOD, GPX, GPX/Protein, TAC, total protein, sulfhydryl groups (SH, SH/Protein), ceruloplasmin (CER), total oxidant status (TOS), lipofuscin (LF), and malondialdehyde (MDA) — which, as a group, show predominantly inverse directional associations with both BMI and idiopathic infertility factor. This pattern is consistent with progressive attenuation of the primary antioxidant buffering capacity of the follicular microenvironment under conditions of elevated body mass and idiopathic reproductive failure (23, 36, 62).

The inverse association of MnSOD with both BMI and idiopathic infertility is particularly noteworthy given its role as the primary mitochondrial antioxidant. Heng et al. (2024) demonstrated that obesity induces mitochondrial damage through excess ROS generation, partly by impairing MnSOD-mediated dismutation of superoxide radicals within the mitochondrial matrix (23). Further studies emphasized that MnSOD is essential for regulating mitochondrial function in adipocytes, and that alterations in its expression — whether genetic, diet-induced, or tissue-specific — carry far-reaching consequences for metabolic and redox homeostasis (63, 64). In the context of follicular biology, where oocytes depend critically on mitochondrial ATP production for spindle assembly and chromosomal segregation, reduced MnSOD activity in the follicular microenvironment may reflect impaired granulosa cell mitochondrial function and could contribute to compromised oocyte developmental competence (65–67). Consistent with this interpretation, a study using an equine obesity model reported reduced expression of both GPX1 and SOD2 in granulosa cells of obese animals, accompanied by elevated intracellular ROS production, and demonstrated that nutritional supplementation normalizing this profile was associated with improved oocyte quality indicators (68).

The inverse association of GPX with idiopathic infertility (posterior probability 0.9995) and BMI (0.9955) is consistent with findings from studies comparing antioxidant enzyme activities across infertility etiologies. Previous studies have reported significantly altered activities of CAT, GR, and GPX in the FF of women with different reproductive conditions, including idiopathic infertility, underscoring the etiological specificity of enzymatic redox disturbances in this compartment (61, 69). The concurrent reduction of GPX and MnSOD in Phenotype 1 may reflect coordinated dysregulation of the mitochondrial–cytoplasmic antioxidant axis rather than isolated enzymatic deficiencies. MnSOD represents a major mitochondrial superoxide-scavenging enzyme, whereas GPX participates in downstream peroxide detoxification; therefore, their parallel attenuation may indicate impaired coupling between mitochondrial ROS generation and cytoplasmic peroxide clearance (9). Potential upstream regulators include mitochondrial dysfunction and impaired oxidative phosphorylation, altered SIRT1/PGC-1α/FOXO3 signaling affecting mitochondrial biogenesis and antioxidant responses, and reduced Nrf2-mediated transcriptional activation of antioxidant enzymes (70). This interpretation is supported by evidence from cumulus cells showing that impaired mitochondrial activity is associated with reduced mtDNA copy number, lower ATP production, down-regulation of oxidative phosphorylation-related genes, and decreased reproductive capacity (14). Thus, the observed co-suppression of MnSOD and GPX may represent a coordinated disturbance of mitochondrial redox regulation within the follicular microenvironment rather than two unrelated enzymatic changes (14).

However, the co-clustering of TOS and LF — markers of oxidant status and cumulative oxidative damage, respectively — within Phenotype 1, both showing inverse associations with BMI, requires careful biological interpretation. At the systemic level, obesity is generally associated with a pro-oxidant state; for example, elevated total oxidant status and reduced antioxidant enzyme activities have been reported in obese individuals, supporting the pro-oxidant character of excess adiposity in peripheral tissues (71). The inverse direction of TOS and LF observed in follicular fluid in the present study should therefore not be interpreted as contradicting the systemic oxidative stress literature, but rather as suggesting compartment-specific redox organization within the follicular microenvironment. Follicular fluid oxidative markers do not necessarily mirror serum concentrations from the same patient, and the follicle maintains active antioxidant mechanisms aimed at protecting the oocyte from oxidative injury (32).

In this context, the concurrent inverse associations of TAC, SOD, MnSOD, GPX, TOS, and LF within Phenotype 1 may indicate a coordinated reduction in both antioxidant buffering and measurable oxidant-related activity in the follicular compartment. This pattern may reflect altered redox turnover, reduced antioxidant–oxidant cycling, or impaired follicular metabolic activity under conditions of increased BMI, rather than a simple decrease in oxidative stress. Thus, the inverse association between BMI and TOS/LF appears to represent a compartment-specific and directionally divergent follicular redox response, consistent with the broader polarization identified in this study.

Progressive depletion of antioxidant capacity in FF — as evidenced by the concurrent inverse associations of TAC, SOD, MnSOD, and GPX in the same cluster — may contribute to lower measurable TOS in a compartment-specific manner by limiting the enzymatic and non-enzymatic reactions that generate oxidant byproducts within this compartment (8, 72, 73). Lipofuscin (LF), in turn, is a marker of cumulative cellular oxidative damage distinct from acute ROS generation: it accumulates intracellularly as a product of the incomplete lysosomal degradation of oxidized proteins and lipids, and its presence in FF likely reflects its release from adjacent granulosa cells undergoing senescence or apoptosis (74, 75). The inverse association of follicular LF with BMI may thus indicate that higher body mass, while inducing systemic oxidative stress, is associated with altered granulosa cell biology — potentially reflecting impaired cellular senescence pathways or accelerated cell turnover — rather than a straightforward increase in cumulative oxidative damage within the follicular compartment. This interpretation is consistent with recent data linking obesity to disrupted granulosa cell mitochondrial function and ovarian cellular senescence (76). The compartment-specific behavior of both TOS and LF underscores the importance of interpreting FF-derived oxidative biomarkers within the unique biological context of the follicular microenvironment rather than by direct analogy to systemic markers.

Two additional markers in Phenotype 1 require specific comment. MDA, despite being the canonical terminal product of lipid peroxidation, showed no statistically significant associations with any of the four clinical factors (Boolean posterior indicators: 0.71, 0.17, 0.82, 0.97; all coded 0). Its assignment to Phenotype 1 thus reflects geometric proximity in taxonomic space to other markers in this cluster rather than a clear directional effect of its own. MDA may be considered a taxonomic ‘passenger’ of this phenotype, and its interpretation should be explicitly decoupled from the dominant signal driving the cluster. A similar caveat applies to CuZnSOD, whose posterior probabilities for all four associations fell near 0.5 (0.96, 0.57, 0.50, 0.52 — all coded 0), indicating the absence of any significant directional effects. The cytoplasmic isoform SOD1 may be subject to different regulatory mechanisms than the mitochondrial MnSOD, potentially explaining why these two isoforms diverge in their responsiveness to the clinical factors examined. This interpretation is biologically plausible, as the transcriptional regulation of SOD1 and SOD2 differs substantially, with the former being less responsive to oxidative stress-induced upregulation (77–79).

### 4.3 Phenotype 2: compensatory upregulation of secondary detoxification enzymes

Phenotype 2 encompasses six markers — GR, GR/Protein, CAT, CAT/Protein, GST, and GST/Protein — which, as a group, showed direct associations with both BMI and idiopathic infertility. These enzymes represent the secondary tier of cellular antioxidant defense: catalase eliminates hydrogen peroxide generated downstream of superoxide dismutation; glutathione reductase regenerates reduced glutathione from its oxidized form, sustaining the glutathione redox cycle; and glutathione S-transferases catalyze the conjugation of electrophilic substrates — including products of lipid peroxidation — with glutathione for detoxification and export. The simultaneous suppression of Phenotype 1 markers and upregulation of Phenotype 2 markers with increasing BMI and idiopathic infertility factor therefore suggests a polarized redistribution of antioxidant enzymatic activity rather than a simple global oxidative or reductive shift.

A mechanistic framework for this compensatory upregulation is provided by the nuclear factor erythroid 2-related factor 2 (NRF2) signaling pathway. Under conditions of chronic oxidative stress — such as those characterizing obesity-related metabolic dysregulation — NRF2 dissociates from its inhibitory partner KEAP1, translocates to the nucleus, and binds to the antioxidant response element (ARE), activating transcription of a broad set of cytoprotective genes including glutathione S-transferases (GSTs) and catalase (CAT) (77, 78, 80, 81). The Nrf2 pathway is described as a pivotal modulator of antioxidant signaling in adipose tissue, with increasing evidence of its involvement in obesity-related metabolic disorders. Importantly, NRF2 target gene activation is also a known response to chronic low-grade inflammation through crosstalk with NF-κB signaling, a pathway consistently activated in adipose tissue of obese individuals (82–84). The expression of these secondary detoxification enzymes in granulosa cells surrounding the oocyte may thus reflect an adaptive cellular stress response that is propagated into the follicular fluid.

However, Phenotype 2 is not internally homogeneous, and two observations require specific discussion. First, and most methodologically important, the absolute activity of GR showed no statistically significant directional associations with any clinical factor (posterior probabilities: 0.086, 0.269, 0.111, 0.321 — all coded 0), whereas GR normalized to protein concentration (GR/Protein) did show a significant direct association with both idiopathic infertility and, at near-threshold probability, with BMI. This discordance implies that the observed increase in normalized GR activity reflects compositional changes in total FF protein rather than, or in addition to, genuine upregulation of GR enzymatic activity per se. Decreasing total protein in FF with increasing BMI — potentially reflecting altered follicular permeability or dilution effects — could arithmetically elevate the normalized ratio even in the absence of true enzymatic induction. This finding has methodological implications for future studies: normalization of enzyme activities to protein concentration in follicular fluid should be reported alongside absolute values, as the two may convey qualitatively different biological information.

Second, GST displayed an inverse association with male factor infertility (posterior probability 0.9976, coded −1) as its sole statistically significant directional effect, which diverges from the direct associations with BMI and idiopathic infertility driving the overall characterization of Phenotype 2. This etiological specificity of GST is noteworthy and biologically plausible: follicular GST activity may be modulated by hormonal and inflammatory signals that differ systematically between infertility etiologies. Patients recruited for male factor infertility presumably have a largely unperturbed ovarian and follicular environment, potentially establishing a different baseline for enzymatic activity in granulosa cells. Supporting this interpretation, (Collodel et al., 2023) demonstrated etiology-specific differences in catalase and glutathione reductase activities across women with idiopathic infertility, endometriosis, and diminished ovarian reserve, confirming that the enzymatic composition of follicular fluid reflects the specific pathophysiological context underlying infertility. The current finding of a GST signal associated with male factor — in the opposite direction — introduces internal heterogeneity into Phenotype 2 that should be acknowledged as a limitation of the purely taxonomic classification approach employed here.

### 4.4 Redox polarization as a shared pathophysiological mechanism of BMI and idiopathic infertility

A central and clinically meaningful observation of this study is that the statistically significant inter-phenotype difference in directional associations was restricted to idiopathic infertility (Fisher’s exact test, p = 0.0097) and BMI (p = 0.0177), while ovarian factor and male factor infertility showed no significant overall effect on the global enzymatic pattern (p = 0.3148 and p = 0.2459, respectively). This selectivity argues against a non-specific effect of inflammation or hormonal stimulation on the redox system, and supports the interpretation that BMI and idiopathic infertility share biological pathways specifically affecting the structured organization of the follicular antioxidant network.

The mechanisms linking obesity to follicular redox dysregulation are multifaceted. Heng et al. provided a comprehensive account of how obesity-induced disruptions of the hypothalamic–pituitary–ovarian axis impair granulosa cell function and alter the biochemical composition of FF (23). Chronic adipose tissue-derived inflammatory signaling via TNF-α and IL-6 further compounds these effects by sustaining a pro-inflammatory state that disrupts cellular homeostasis within the follicular microenvironment (26). Alterations in leptin and adiponectin signaling further modify intrafollicular steroidogenesis and cellular metabolism. Importantly, these systemic metabolic perturbations are reflected at the level of follicular fluid composition, which differs substantially between women of normal weight and those with excess adiposity, as evidenced by alterations in both lipid and enzymatic components of this compartment (4, 85). The present data suggest that these perturbations manifest at the enzymatic level as a polarized redistribution of antioxidant activity: primary enzymatic defenses (SOD, GPX, MnSOD) are attenuated — a pattern consistent with reports of reduced SOD and CAT activity in FF of women with impaired reproductive outcomes while secondary detoxification capacity (CAT, GR, GST) is upregulated, potentially as a compensatory response (86). This pattern could be interpreted as a transition from a physiologically regulated antioxidant state to an adaptive stress-response state — a shift that, if sustained, may impair the finely tuned redox signaling required for normal oocyte maturation and ovulation (23).

The finding that idiopathic infertility is associated with an identical pattern of redox polarization as BMI is of considerable diagnostic and conceptual significance. Idiopathic infertility is a diagnosis of exclusion affecting approximately 30% of infertile couples, defined by the absence of detectable pathology on standard clinical and laboratory assessment (2). The existence of a distinctive enzymatic phenotype profile in the FF of women with idiopathic infertility supports the hypothesis that this condition reflects genuine, albeit biochemically subtle, pathological alterations in the follicular microenvironment rather than a true absence of pathology. This interpretation aligns with prior evidence from lipidomic and proteomic studies demonstrating metabolic signatures specific to idiopathic infertility in FF that are absent in women with male factor infertility as controls (36, 61). It also corroborates observations that follicular fluid from women with unexplained infertility exhibits an oxidative imbalance and reduced melatonin concentrations, suggesting compromised intrafollicular antioxidant homeostasis (87, 88). The current study extends these findings by providing the first evidence that this imbalance takes a specific polarized, system-level form, differentiating idiopathic infertility from male factor and ovarian aetiologies at the level of enzymatic network organization.

The overlap between the redox polarization patterns associated with BMI and idiopathic infertility raises the possibility that elevated body mass may, in some patients, contribute mechanistically to the development of idiopathic infertility through follicular redox reorganization. While the study design does not permit causal inference, and the effects of BMI and idiopathic factor were evaluated as independent predictors in the multivariable model, the convergence of their effects on the same enzymatic polarization axis is suggestive of shared downstream pathways. The degree to which obesity-related metabolic and inflammatory signaling may generate a biochemical environment indistinguishable from idiopathic infertility on standard assessment — but detectable through FF redox phenotyping — merits investigation in larger, purpose-designed cohort studies.

### 4.5 Strengths, limitations and future directions

The methodological strength of this study lies in the integration of probabilistic modeling with direction-based pattern recognition and unsupervised taxonomic classification applied to follicular fluid enzymatic data. The Boolean posterior indicator framework — in which the direction and stability of each regression coefficient is quantified across MCMC iterations — provides a nuanced representation of association strength and directionality that exceeds what is achievable with conventional point estimates and p-values. The repurposing of the Marczewski–Steinhaus taxonomic metric to quantify inter-enzyme similarity based on multivariable association patterns, rather than raw biochemical concentrations, constitutes an original analytical contribution that may be applicable to other systems biology investigations of complex biological fluids.

With regard to missingness among oxidative biomarkers, the proportion of missing data was moderate (8.4%) and resulted primarily from unavailable paired follicular fluid samples rather than assay-specific measurement failures. Because missing observations were handled as latent variables within the Bayesian MCMC framework, all available patient information contributed to parameter estimation. Nevertheless, if the mechanism generating missing values differed systematically from the observed data, some degree of residual uncertainty may remain. Therefore, the identified phenotype structure should be interpreted as robust within the present dataset but warrants confirmation in independent cohorts.

Several limitations of the present study should be explicitly acknowledged. First, the study included 77 patients undergoing IVF treatment, which represents a moderate sample size. Although the paired assessment of follicular fluid from both ovaries increased the amount of biochemical information available for system-level modeling, the findings require validation in larger and independent cohorts. Second, the study population was limited to patients undergoing assisted reproduction, which may restrict the generalizability of the results to broader reproductive or non-IVF populations. Third, the present analysis was cross-sectional; therefore, temporal dynamics, intra-cycle stability, or longitudinal reproducibility of the identified redox phenotypes could not be evaluated.

Several potential clinical confounders may influence follicular fluid oxidative biomarkers, including maternal age, ovarian reserve, infertility etiology, metabolic status, and ovarian stimulation characteristics. These factors were addressed at the analytical level by including age, AMH concentration, duration of hMG/FSH stimulation, IVF attempt number, and major infertility-related diagnoses in the initial Bayesian multivariable model. In the final directional structure, however, age, AMH concentration, stimulation duration, IVF attempt number, tubal factor, and endometriosis were not retained as stable phenotype-level drivers, whereas BMI and idiopathic infertility showed the most consistent associations with redox phenotype polarization. This suggests that the identified structure was not primarily explained by age, ovarian reserve, or stimulation duration within the present dataset. Nevertheless, residual confounding cannot be excluded. In particular, more detailed stimulation-related parameters, such as total gonadotropin dose, protocol subtype, estradiol response, number of follicles, follicular size distribution, and trigger type, were not modeled as independent determinants of redox phenotype organization.

Another limitation is the absence of direct embryological and reproductive outcome data in the present analysis. Outcomes such as fertilization rate, embryo development, blastocyst formation, implantation, clinical pregnancy, miscarriage, and live birth were not included; therefore, the prognostic significance of the identified phenotypes cannot yet be established. Nevertheless, previous studies have demonstrated associations between follicular fluid oxidative balance and IVF outcomes, supporting the potential biological relevance of the polarized enzymatic patterns identified in the present analysis (6, 89). Moreover, recent systematic evidence indicates that follicular fluid composition differs substantially across reproductive disorders. For example, Moreira et al. (90) showed that in women with PCOS undergoing IVF, follicular fluid differs from that of normo-ovulatory women mainly in molecular classes related to oxidative stress, inflammatory biomarkers, growth factors, and hormones. These findings support the broader concept that reproductive disorders may be associated with disorder-specific alterations in follicular biochemistry. This interpretation is further strengthened by the recent global consensus proposing the term polyendocrine metabolic ovarian syndrome (PMOS) instead of PCOS, emphasizing that the condition represents a multisystem endocrine–metabolic–ovarian disorder rather than an isolated ovarian phenotype (91). At the same time, both the systematic review and the evolving nomenclature highlight the need for future follicular fluid studies based on well-characterized metabolic, endocrine, inflammatory, and ovarian subgroups.

Future research should aim to replicate the Bayesian Directional Taxonomic Clustering approach in larger, independent, and prospectively designed cohorts. Such studies should incorporate both enzymatic and non-enzymatic redox markers, more granular ovarian stimulation data, detailed metabolic and inflammatory profiling, and direct measures of oocyte quality and embryo development. The integration of follicular fluid proteomics, metabolomics, and granulosa or cumulus cell transcriptomics with the enzymatic phenotype framework proposed here could provide mechanistic insight into the biological processes driving redox polarization. From a clinical perspective, the identification of redox phenotypes in follicular fluid may offer a potential route for stratifying patients according to their follicular biochemical profile, particularly in metabolically vulnerable groups such as patients with elevated BMI, idiopathic infertility, or defined endocrine phenotypes. In the future, such stratification could help identify patients who may benefit from preconception or pre-IVF interventions aimed at improving follicular redox homeostasis, including weight optimization, metabolic management, lifestyle modification, or carefully targeted antioxidant strategies. However, antioxidant supplementation should not be interpreted as a universal intervention, as physiological ROS signaling is required for follicular maturation, ovulation, and oocyte competence. Therefore, the clinical utility of redox phenotype-based stratification must be assessed in prospective, outcome-linked studies before individualized antioxidant or metabolic interventions can be recommended in routine ART practice.

## 5 Conclusions

The present study demonstrates, for the first time using an integrated Bayesian–taxonomic analytical framework, that oxidative enzymes in human follicular fluid form two structurally distinct and functionally antagonistic phenotypic clusters. Phenotype 1 included enzymes representing the primary antioxidant defense system, whose activity tended to decrease with increasing BMI and idiopathic infertility. In contrast, Phenotype 2 comprised enzymes involved in secondary detoxification pathways, which showed an opposite pattern and tended to increase in association with the same factors. Together, these findings suggest that obesity and idiopathic infertility are associated not with uniform oxidative changes, but with a polarized reorganization of the follicular redox environment. The selective polarization of this enzymatic landscape by BMI and idiopathic infertility, but not by ovarian or male aetiologies, points to a shared pathophysiological mechanism that reorganizes, rather than uniformly depletes, the follicular antioxidant network. These findings challenge the prevailing single-biomarker paradigm in reproductive oxidative stress research and support a systems-level interpretation of FF biochemistry. The described enzymatic phenotyping approach may ultimately contribute to a more nuanced biological characterization of idiopathic infertility and obesity-related reproductive dysfunction, with potential implications for patient stratification and targeted therapeutic strategies.

## Data Availability

The original contributions presented in the study are included in the article/supplementary material. Further inquiries can be directed to the corresponding author.
